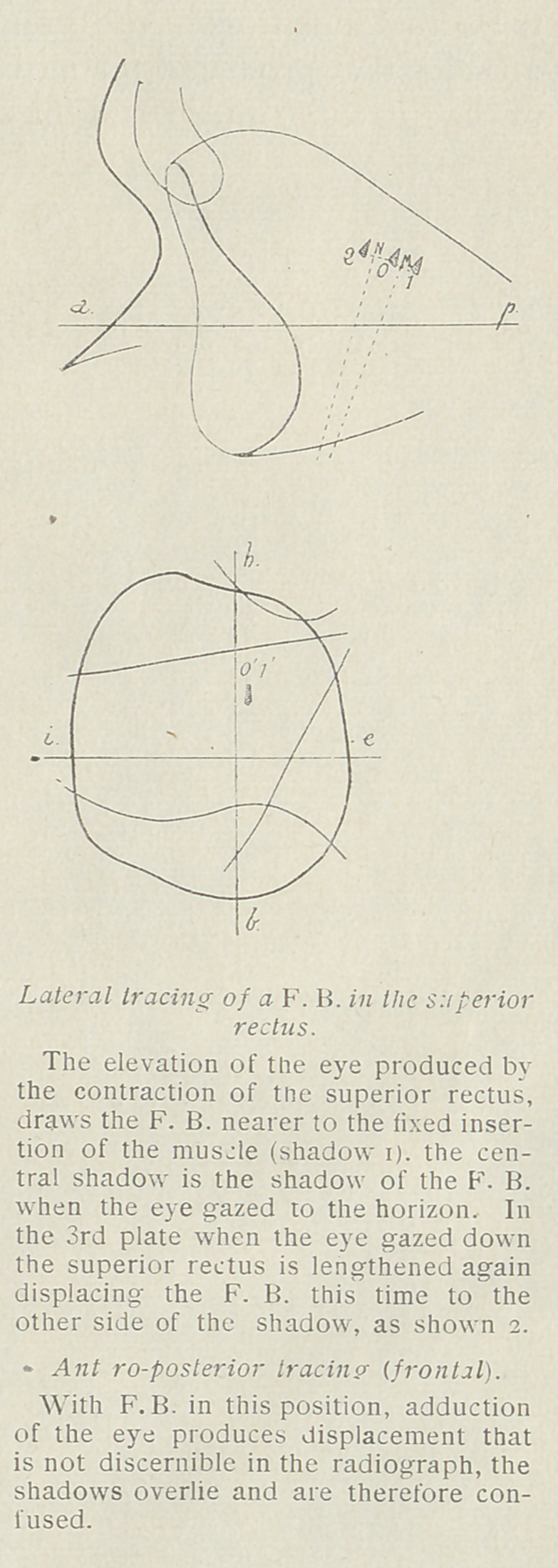# X-Ray Examination for Foreign Bodies in the Eye and Their Localization

**Published:** 1918-04

**Authors:** 


### RADIOLOGICAL

***X-Ray Examination for Foreign Bodies in the Eye and Their Localization.*** By J. Belot, Président de la Société de Radiologie, and H. Fraudet, Agrégé de Physique. Trans. and Abs. from the *Journal de Radiologie*, January-February, 1917. by Harold C. Gage, Chef du Service Radiologique, Hôpital Militaire V. R. 76, Ris-Orangis, France. Plates are reproduced by the courtesy of the *Journal de Radiologie*.

The observation and localization of foreign bodies in the eye are the most important and most difficult of radiological operations.

A tiny foreign body which might be of no importance anywhere else, if in the eye, must be found and localized with great accuracy: or, if an observation gives negative results, there must not be a shadow of a doubt, for, if overlooked, such a particle may by sympathetic ophthalmia occasion the loss of the other eye.

Fluoroscopy may be useful in following the movements of the foreign body while the eye is being rotated, but, although much useful information can be obtained in this manner, it should never be relied upon for a negative diagnosis.

In radiography, a very rigorous technique is necessary, as is also a minute examination of the plates. Radiographs of the region of the eye are complicated by numerous overlying shadows of bony structures which cannot be obviated by any artifice; this fact adds greatly to the difficulty of observation when fragments of microscopical proportions must be detected. The technique of observation requires immobilization of the head and a powerful installation.

The problem is further complicated by foreign bodies of relative transparency to the X-rays, such as, aluminium, glass, etc.

Other methods are sometimes used, as, for instance, the " double image " or " stereoscopic " techniques, but in the author's opinion, these should be reserved only for cases in which the eye has lost all movement.

For the practice of the method described in the following paragraphs, sight must have been preserved in one eye, and the wounded eye must have retained its mobility.

*Description of the Method and Technique.* The method consists of two steps — Examination and Localization. Whenever possible the radiologist should be in possession of the ophthalmogist's report, which may render some assistance.

A lateral examination is made first, and the whole area studied with a very small diaphragm opening. Foreign bodies may be found in other parts of the face and head, and it is easy to determine roughly the position of all foreign bodies and thus prevent confusion if there are more than one in the region.

The head is next adjusted in a lateral position for the examination of the eye in question. With the screen in contact with the injured eye side of the head, the tube is adjusted so that the normal ray shall pass through the orbital cavities: this position is easy to identify by the bright, almost oval, patch just posterior to the nasal bones.

If a foreign body is discovered here, it remains to determine whether it is in the globe. For this purpose, the patient is asked to look up and then down, for in this manner the movement of the foreign body may be interpreted. If moving in the same direction as the eye, the foreign body will be in the anterior hemisphere; but if it moves in the opposite direction, the foreign body will be in the posterior hemisphere. Further diffe-rentiation is necessary, because a foreign body in the muscles producing the movement of the eye, will also be displaced. This point is dealt with later. Care should be taken to exclude the possibility of foreign bodies in the eye-lids. Should the shadow of a foreign body be seen very anterior, moving rapidly as the patient opens and closes the eye, this location may be suspected. The parts may be immobilized individually during the screen examination; from such procedure a diagnosis can be formed.

Much useful additional and corroborative information can be obtained by a supplementary anterior-posterior examination.

*Radiography.* — For the exact localization 5 radiographs will be required: 3 lateral and 2 A. P.

It is assumed, as previously stated, that sight is preserved in one eye, and that the wounded eye has retained its mobility.

It is agreed that the eye may be regarded as a sphere, and that its movements are those of rotation about its center. Since the center remains fixed, a foreign body in the eye will make movements which are definitely related to the rotation of the eye. The comparison and study of successive radios between which the eye has been rotated in a definite direction, will give data upon which an exact localization can be made.

If the foreign body performs a rotation around the same axis equal to the rotation of the eye, the foreign body is certainly in the eyeball or in a part of the muscle. If the displacement is not about the same axis, a careful study will show whether it is in the soft parts or in a muscle, and ultimately in which muscle it is situated.

It is assumed in what follows that, should the injured eye not have retained sufficient sight, the two eyes will make identical movements.

*Making the Lateral Radiographs.* — For this purpose it is desirable to use a small tunnel for the plate so that the plate can be easily changed while the head is kept immobilized. Small plates (9 × 12 cm.) are sufficient. Across the opening under which the plate slides, a fine wire is placed, for it is necessary to record on the plate the horizontal (in the anatomical position) equator of the eye. The head is adjusted on the tunnel in such a manner that the metal wire coincides as a parallel line to an imaginary line passing through the center of the cornea and back through the central axis of the eye. While the patient gazes towards the horizon, this plane is recorded on the plates.

The tube should be centered above at a sufficient distance so that the resulting radiograph of the globe may be considered actual[1]. The normal ray should pass through the central axis of the eye and be at right angles to the plate.

With the patient, tube, and plate so arranged, three radiographs are now made, with the head immobilized; in the first plate (to be marked O) the patient's gaze is directed to the horizon; in the second plate (marked "up") the gaze is directed upwards; and in the third plate the gaze is directed downward and the plate marked accordingly.

*The Anterior-posterior Radiographs.* — Two fine wires are adjusted on a

1. For the lateral radiograph, anticathode to plate 800 mm. gives a maximum error of 1 mm.: for the anterior, anticathode to plate 650 mm. gives also a maximum error of 1 mm.

frame so that their intersection shall be vertically over the center of the cornea, the wires coinciding with the horizontal and vertical equators of the eye. The patient gazes straight in front of him, that is in this case, to the ceiling. The tube must be centered so that the normal ray shall pass through the intersection of the cross wires. With the patient so disposed, and the tube and plate so arranged, the first plate is exposed. For the second exposure, the patient is directed to gaze with the wounded eye turned inward (adduction). From the study of the five plates thus made the diagnosis can be determined.

The first step towards diagnosis is to make tracings from the lateral Radiographs: from these, one composite tracing is made. From plate O the outline of the bony skeleton of orbit and of the metal wire is drawn on transparent paper. The foreign body is also traced; this should be done with special attention to any orientation it possesses. By carefully superimposing the tracing on 1 and 2 the foreign body shadows from those plates are added.

Composite tracing " lateral " with center of the eye projected not corresponding to the line used to show the *a. p.* axis of the eye and produced by the metal wire. The wire was not correctly adjusted for the *a. p.* axis must necessarily pass through the center of the globe.

The same procedure is carried out in producing the anterior-posterior tracings. These tracings may be called lateral and frontal.

*Study of the Tracings.* — It is possible that the shadows of the foreign body may completely overlie. They may overlie in one and be neatly separated in the other, or be separated in both tracings.

A foreign body that has not moved at all is either *not in the eye or the muscle* or else *it is in the center of the eye.* This is an important point which must never be overlooked : it may mean that a tiny foreign body is located in the vitreous humor or is adherent to the posterior surface of the crystalline lens.

If the foreign body is in the center of the globe, its position in the lateral tracing will be slightly anterior to the shadow of the malar border of the orbit, and also near to the wire that materializes the anterior-posterior or horizontal axis of the eye , and on the frontal tracing it will coincide, or nearly so, with the center of the cross wires. This question will arise only when the foreign body is very tiny and circular in shape ; otherwise it will be possible to follow the orientations of the foreign body in the changes of position.

*Geometrical Construction.* In lateral tracings in which the foreign body is shown to have moved, producing three successive shadows, the proceedings are as follows:

Two fine lines are drawn connecting the 3 foreign-body shadows, using the same point of orientation of the foreign body. From the centers of these two lines, perpendicular lines are elevated, their intersection marking the center of a circle, the circumference of which passes through the foreign body.

In this manner the center of the globe is materialized. If this point falls just anterior to the malar border of the orbit, the foreign body is in the globe, and its position can be given in two directions, and the third obtained from the antero-posterior tracings. If the intersection falls remote from the malar border and from the antero-posterior or horizontal plane projection, the foreign body is not in the globe but in one of the muscles.

To ascertain whether the movement of the foreign body corresponds to the rotation of the eye, a long ruler fitted with a movable electric lamp is placed at a known distance from the patient and used to direct his gaze when taking the lateral plate, and the displacements above and below the central or horizontal position are recorded. With this information, using cm. to represent a meter, the angle the eye has turned can be reconstructed on the lateral tracing, showing definitely whether the foreign body has swept the same angle as the eye.

*Foreign Bodies in the Muscles of the Orbit.* In those tracings that show the foreign body to have suffered movement, and yet in which the center of the circle, of which the foreign body shadows represent points on the circumference, does not fall in the position indicated as the center of the globe, a little study will reveal the actual position of the body, if the observer bears in mind the muscles that produced the movements of the eye in the production of the plates. Interpretation is thus made comparatively simple.

*Le Gérant* : O. Porée. Paris — Imp. Lahure, 9, rue de Fleurus.

## Figures and Tables

**Figure f1:**
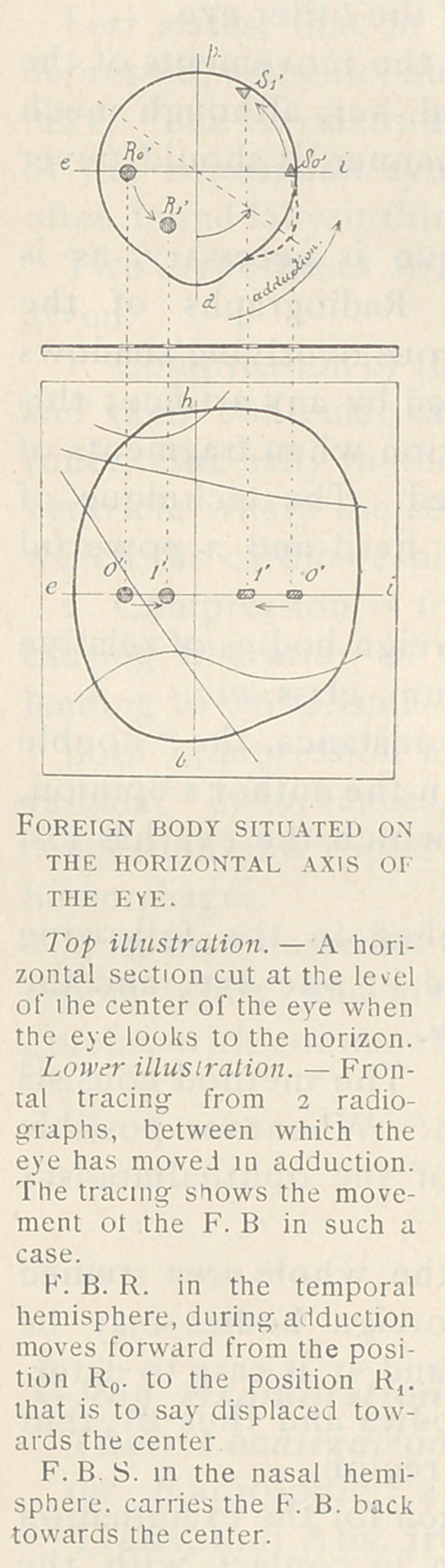


**Figure f2:**
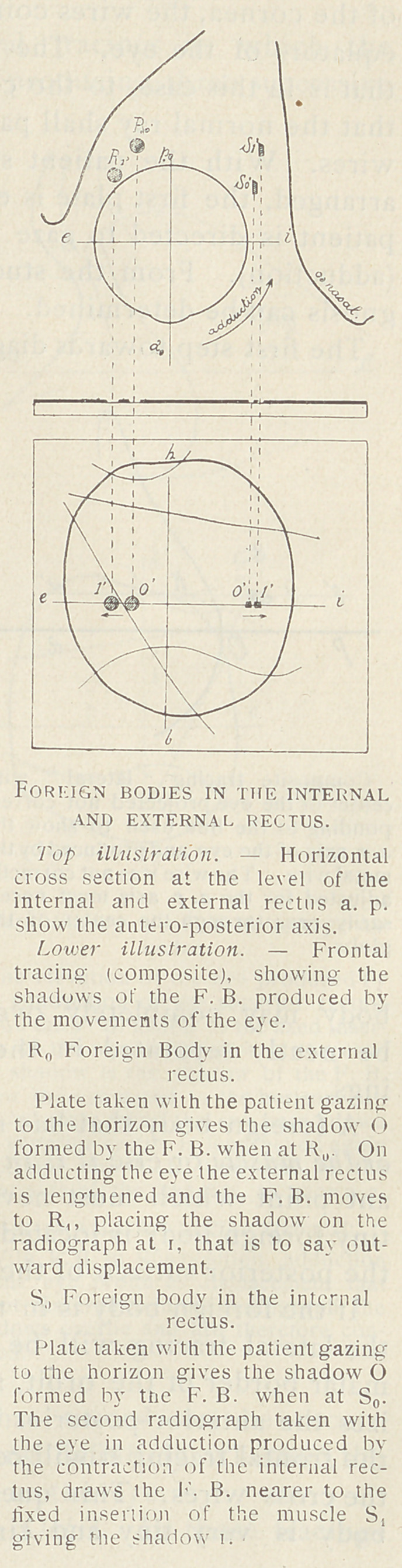


**Figure f3:**
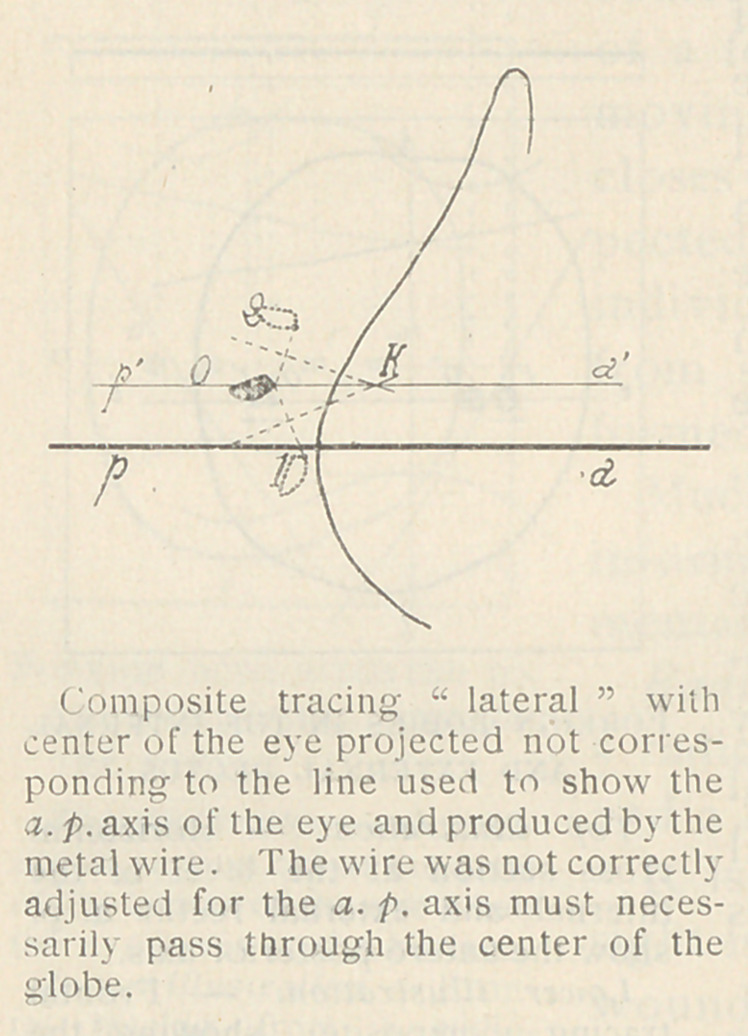


**Figure f4:**